# The Breakdown of the Hospital System

**Published:** 1889-03-30

**Authors:** Lawson Tait

**Affiliations:** Surgeon to Birmingham and Midland Hospital for Women


### THE BREAKDOWN OF THE HOSPITAL SYSTEM.

By Mr. LAWSON TAIT, F.R.C.S., Surgeon to Birmingham and Midland Hospital for Women.

*(Continued from page 393.)*

IN the first place, I am entirely in accordance with Dr. Sutherland, that the system of government is not in harmony with modern ideas, and that in many centres the hospital accommodation is not adequate; this latter result being due to the unsatisfactory financial position of the institutions. But I do not think that the author takes into consideration in his proposed remedies that many of the difficulties are purely of local origin; this, for instance, is clearly the case in Birmingham, which he cites as a model example of hospital management. What these local conditions are I do not propose to indicate, but from a long experience of hospital work here, I may say that we quite well understand them. The remedy which Dr. Sutherland suggests for these troubles would be totally inadequate—in fact it would be detrimental; that is, the creation of a central authority, having anything to do with a Minister of Health. The hospital must be managed by local persons entirely, and upon local requirements: it is eminently a case for Home Rule.

That all hospitals should be free, and that admission should be regulated only by the requirements of the cases, are proposals which I do not stop to argue, because they must and will be universally accepted. The system of admission should be based entirely, as I have said, upon the requirements of each case, and the medical authorities alone should be made responsible for the admissions and for the discharges, the committee having no duty in this matter beyond that of seeing that the medical officers do not either neglect or malvert their privilege.

One great fault untouched by Dr. Sutherland in hospital management is that the medical officers are appointed for life, and that they are therefore far too apt, especially when allowed to sit upon the boards of management of the institution, to acquire a freehold right over the benefits in their possession. They would be kept far better to their work if they were subject to periodical election, it being quite an understood thing that the re-election would follow in the event of the management being satisfactory. This is so in my own case; I am re-elected to my hospital position every year, and would be certain of non-election if I conducted myself as many of the hospital officers round me do.

The financial question of hospitals is a very difficult one, and here again local influences are at work. An old, well-established hospital, firmly fixed in the affections of the people, may be full of abuses, and yet its financial position will be quite assured by reason of popular familiarity with its position, with its name and external appearance. A new institution, doing far better work, relatively far more free from abuse, will often have the utmost difficulty to achieve an existence. A great remedy for this, in my eyes—in fact a remedy for many of the hospital abuses—will be found in the Hospital Saturday collection made in workshops, increasing every year, and involving, as a matter of course, that those who contribute will have a share in the management of the institutions. This we find to be the case in Birmingham, and the artisan class, for whom the hospital is especially conducted, will be found year by year increasing their payments towards the hospitals, and having an increased interest in the distribution of the funds. This will prove a much better arrangement than the ideal central authority.

Upon the financial question raised in Dr. Sutherland's paper I have very little to say, except that the establishment of convalescent homes and sanatoria follow, as a matter of course, to everyone who is acquainted with the necessities of hospital detail.

That the hospitals at present attached to workhouses and poorhouses should be divorced from them I cannot regard as necessary at all, and I do not see why the hospital system should be regarded as separate from the workhouse system. They are both charitable, with this temporary difference, that the workhouse and parochial relief are branded with a disgrace which ought to form no part of it, for the truest and best forms of socialism must regard a pauper as a citizen unfortunately exhausted in the struggle for life, and stranded in a position not quite satisfactory; but why he should be branded with disgrace on account of his misfortune, and because he elects not to starve, I, for one, cannot understand, and therefore I am disposed to do everything I can to raise the regard which we must have towards workhouses and poorhouses. I would construct a workhouse hospital alike in building as in executive management with our hospital system, and instead of further divorce would rather favour a closer union. Therefore it is that I have recently proposed that our new workhouse hospital in Birmingham should be provided with a consulting staff, just as our other hospitals have. By this I do not mean that they should be provided merely with men who have retired from an acting staff, and, by reason of seniority, are perhaps hardly fit for further duty, but that an actual consulting staff should be elected and properly used when required. This alone would raise the position of the workhouse hospital in the estimation of the public and of professional men alike more nearly to the position of the general public hospital. I regret to find that my intention has been entirely misunderstood and misrepresented by a leading medical journal, and I sincerely trust that the management of the workhouse hospital will have no such misunderstanding on the subject.

